# Simultaneous determination of *cis*- and *trans*-palmitoleic acid in rat serum by UPLC–MS/MS

**DOI:** 10.1038/s41598-022-20739-x

**Published:** 2022-10-05

**Authors:** Wenwen Huang, Yiping Zhang, Liping Zhong, Chunlong Sun, Zaiwang Zhang

**Affiliations:** 1 Binzhou Key Laboratory of Chemical Drug R&D and Quality Control, College of Biological and Environmental Engineering, Shandong Provincial Engineering and Technology Research Center for Wild Plant Resources Development and Application of Yellow River Delta, Binzhou University, Binzhou, 256603 People’s Republic of China; 2 The Third Institute of Oceanography, Ministry of Natural Resources, Xiamen, 361005 People’s Republic of China; 3 Technical Center of Xiamen Entry-Exit Inspection and Quarantine Bureau, Xiamen, 361026 China

**Keywords:** Biochemistry, Drug discovery, Health occupations

## Abstract

Palmitoleic acid, a monounsaturated fatty acid which could affect glucose and lipid metabolism and reduce insulin resistance has two isomers, i.e. *cis*-palmitoleic acid (*c*POA) and *trans*-palmitoleic acid (*t*POA). However, the pharmacokinetic, metabolic transformation and structure–activity relationship of the two isomers have not been reported. A precise and accurate ultra performance liquid chromatography–tandem mass spectroscopy (UPLC–MS/MS) method was developed to determine *c*POA and *t*POA simultaneously. Both the *c*POA and *t*POA were administered i.g. (intragastric gavage) to rats at 75 mg/kg. Serum samples were collected and analyzed for the two isomers by UPLC–MS/MS on a reverse-phase BDS C18 column equilibrated and eluted with water (A) and acetonitrile (B) at a flow rate of 0.3 mL/min. The calibration curves for *c*POA and *t*POA were linear over the range 0.1–12 μg/mL. Analytes were monitored by selected-reaction monitoring in negative electrospray ionization mode. The Tmax of *c*POA was 0.94 ± 0.44 h and the Cmax 8.17 ± 1.97 μg/L, and the Tmax and Cmax of *t*POA were 1.50 ± 0.98 h and 14.77 ± 11.91 μg/L, respectively. AUC_0–24 h_ of *c*POA and *t*POA were 59.45 ± 29.83 and 113.88 ± 72.25 mg/L·h. The method was applied in pharmacokinetic study of *c*POA and *t*POA in rat serum successfully. Besides, the concentrations of *c*POA and *t*POA in rat serums were observed fluctuating with a consistent trend, which may be due to reciprocal bio-convert in the body.

## Introduction

**Figure 1:**
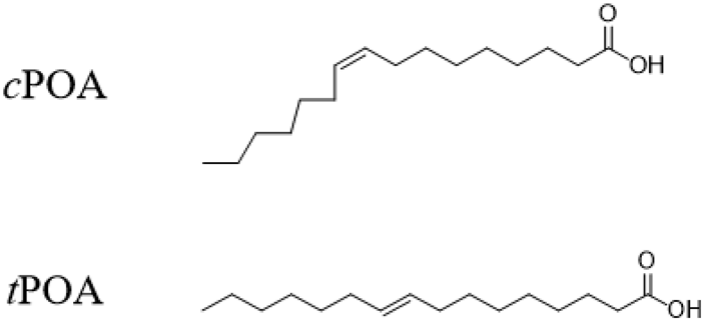
Structures of *c*POA and *t*POA.

Palmitoleic acid (POA, C16:1, n-7) is an omega-7 monounsaturated fatty acid in plant and fish oil^1^. It has two isomers, i.e. *cis*-POA (*c*POA) and *trans*-POA (*t*POA), which are with different space structures (Fig. 1). *c*POA is common in natural POA, which has been widely reported affecting favorably glucose and lipid metabolism through multiple mechanisms^2,3^. *c*POA was observed affecting the key enzymes of blood glucose metabolism, regulating insulin secretion and reducing insulin resistance^4,5^. Orally administered *c*POA induced satiety, enhanced the release of satiety hormones and decreased food intake in mice^6^, as well as reduced body weight, ameliorated the development of hypertriglyceridemia and hyperglycemia, and improved insulin sensitivity^7^. On the other side, *trans*-fatty acid is a subject of ongoing discussions on both suggested positive and negative associations with cardiovascular and metabolic risk factors^8^. Higher *t*POA proportion in plasma phospholipids improved insulin sensitivity or decreased onset of type 2 diabetes disease^9^. *t*POA regulated glycolipid metabolism, closely related to low density lipoprotein^10–12^. It has been used as a biomarker for reducing the risk of coronary heart disease and type 2 diabetes. Most of the studies analysed fatty acids using GC-FID or GC-Ms^13^. The fatty acids samples used in the process requires separation and methylation before the measurement. This may destroy the original molecular structure of fatty acids in the biological matrix, and require big biological sample size or amount than other methods^14,15^. In previous studies, *c*POA was determined in the subcutaneous fat of human body, and *t*POA was determined in the 200 µL plasma of human by GC thorough, aminopropyl SPE columns for the separation (Isolute, Biotage) and transmethylated with methanolic-HCl into fatty acid methyl esters (FAME)^16–18^, but no method was available to determine *c*POA and *t*POA at the same time. So, it’s important to develop a method for the simultaneous determination of *c*POA and *t*POA in biological samples to explore the biological activity, drug delivery systems or regenerative medicine. In the present paper, a quantification method using UPLC–MS/MS to determine *c*POA and *t*POA simultaneously in rat serum was developed. It was proved to be selective and sensitive, with a wide range of detection and low limit of detection, which could be used to ascertain the pharmacokinetics of *c*POA and *t*POA.

## Materials and methods

### Materials and animals

*c*POA and *t*POA were purchased from NU-CHEK (> 99%, Elysian, USA). HPLC-grade formic acid was obtained from Roe Scientific Inc. (Powell, OH, USA). HPLC-grade acetonitrile, methanol and MTBE (methyl tert-butyl ether) were obtained from Merck KGaA (Darm-stadt, Germany). Ultrapure water was from a Millipore Milli-Q system (Millipore Corp., Billerica, MA, USA). The other solvents or reagents were commercially available and reagent grade.

The use of rat approved by the Experimental Animal Ethics Committee of Nanjing University of Traditional Chinese Medicine in accordance with NIH guidelines. Ethical approval number: 201811A027. The authors complied with the ARRIVE guidelines. The 18 male Sprague-Dawley rats weighing 280 ± 20 g were maintained with 12 h:12 h light/dark cycles in a temperature-controlled room throughout the study. Standard rat chow and tap water were supplied ad libitum. Rat serum was collected from vein of rat after narcotized by intraperitoneal injection of pentobarbital sodium (Produlab Pharma B.V., 40 mg/kg).

### Chromatographic and mass spectrometric conditions

#### Liquid chromatography conditions

Analyte separations were performed on an Agilent UPLC-1290 system (Agilent Corp., Milford, MA, USA) using a BDS C18 column (2.1 × 100 mm, 2.1 μm, Thermo Fisher Scientific, USA). The mobile phase was included water (A) and acetonitrile (B) (A:B = 20:80, v/v) at a flow rate of 0.3 mL/min, and the injection volume was 1 μL.

#### Mass spectrometric conditions

Identification of *c*POA and *t*POA in serum samples was conducted using an AB 5500 Q-trap UPLC–MS/MS (AB Sciex, Framingham, MA, USA) equipped with electro spray ionization (ESI). Quantitative analysis of *c*POA and *t*POA were also performed by UPLC–MS/MS. Detection was performed in negative ion mode under the following conditions: curtain gas at 35.0 L/h, and ion source gases at 50 L/h. AB Analyst 1.6.0 software (AB Sciex, Framingham, MA, USA) was used for system control and data acquisition. ESI-MS/MS parameters are shown in Table 1. Detection was performed in the negative ion mode and conditions for *c*POA and *t*POA detection optimized using standards. Product ions obtained from deprotonated molecular ions of *c*POA and *t*POA included three main ions from each compound at m/z 235.2, 126.9, 111.0 and at m/z 234.7, 127, and 111.1, respectively.

**Table 1:** Electrospray ionization ESI-MS/MS parameters for *c*POA and *t*POA.

Analyte	Precursor ion (m/z)	Daughter ion (m/z)	Dwell time (s)	DP (V)	EP (V)	CE (V)	CXP (V)
*c*POA	253.2	235.2	20	− 100	− 8.2	− 25.8	− 11.0
	253.2	126.9	20	− 100	− 8.2	− 31.0	− 11.0
	253.2	111.0	20	− 100	− 8.2	− 29.0	− 11.0
*t*POA	253.2	234.7	20	− 100	− 7.3	− 28.0	− 8.50
	253.2	127.0	20	− 100	− 7.3	− 26.0	− 12.0
	253.2	111.1	20	− 100	− 7.3	− 26.0	− 12.0

### Stock solutions and working solutions

Individual standard stock solutions of *c*POA and *t*POA (2.50 mg/mL, 2.24 mg/mL respectively) were prepared in acetonitrile. These stock solutions were serially diluted with acetonitrile to provide standard working solutions in the concentration range of 0.175–42.0 μg/mL for *c*POA and *t*POA. All solutions were stored at − 20 °C and brought to room temperature before use.

### Calibration standard curves and QC samples

Calibration standard (CS) curves were prepared by spiking 20 μL of the appropriate analyst working solution into 50 μL of blank rat serum. The effective concentrations were 0.1, 0.5, 2.5, 5, 10, 12 μg/mL for *c*POA and *t*POA. QC samples were prepared as compound samples for each concentration at 0.5 μg/mL for *c*POA and *t*POA, and stored at − 20 °C until use. Rat serum samples, serving as QCs, were processed the following sample procedure as for unknown samples.

### Serum sample preparation

50 μL of serum sample (blank, or pharmacokinetics serum sample) in a 2.0 mL centrifuge tube, 60 μL of aqueous solution with formic acid in 5%, 100 μL methanol, and 1250 μL MTBE were added and mixed by vortexing for 3 min. After centrifugation at 18,000×*g* for 10 min, the clear supernatant of 1 mL was extracted to a new centrifuge tube, blowed by flowing nitrogen, and redissolved by 200 μL acetonitrile solution. Centrifugation at 18,000×*g* for 10 min again, supernatant was injected into the UPLC–MS/MS system.

### Method validation

Assay validation performed was based on the currently accepted FDA prescription and per guidelines of the International Conference on Harmonization of Technical Requirements for Registration of Pharmaceuticals for Human Use^19^. Each blank serum sample was processed through the extraction procedure and tested to ensure no rat serum interference with the analyte. While the serum sample preparation was 60 μL of aqueous solution with formic acid in 5%, 100 μL methanol, and 1250 μL MTBE were added and mixed by vortexing for 3 min. After centrifugation at 18,000×*g* for 10 min, the clear supernatant of 1 mL was extracted to a new centrifuge tube, blowed by flowing nitrogen, and redissolved by 200 μL acetonitrile solution.

The determination of the extraction recoveries of *c*POA and *t*POA was at three QC concentrations. And the calculation of the recoveries was by comparing analyte peak area ratios for each analyte in serum samples with those of analytes in the serum matrices by extracting analyte-free serum samples which were prior to chromatography. In extracted rat serum, matrix effects from endogenous substances were presented, which might have caused ion signal suppression or enhancement. Matrix effects at three QC concentrations (0.5, 2.5, and 10 ng/mL) were measured by comparing peak responses of samples post-extraction (A) with that of pure standard solution which contained equivalent amounts of the two compounds (B). The ratio (A/B × 100%) was used to evaluate the matrix effect and the extraction recovery and matrix effect of *c*POA and *t*POA were evaluated simultaneously by the same method.

During sample storage and processing procedures, the stability of *c*POA and *t*POA in rat serum was assessed by analyzing replicates (n = 6) of three QC concentrations. The freeze–thaw stability was determined through three freeze–thaw cycles. All stability testing of QC samples were determined according to calibration curves of freshly prepared standards.

### Pharmacokinetic studies of *c*POA and *t*POA

Male rats (Sprague-Dawley, 280 ± 20 g) were obtained from the Laboratory Animal Center of Nanjing University of Chinese Medicine (Nanjing, China). Animal handling procedures followed standard operating procedure approved by the institutional animal care and use committee. All rats were dosed following overnight fasting except for water ad libitum. For pharmacokinetic studies, 18 male rats were randomly divided into three groups. In the first group, rats were given the same volume of solvent saline intragastrically. Blood samples were collected at the time points of 0, 10, 20, 30, 40, 60 min, and 2 h, 3 h, 6 h, 12 h, 24 h. Rats in the second group were administered i.g. of *c*POA with 75 mg/kg body weight. Serial blood samples were collected in tubes via the orbital venous plexus before and at time points of 0, 10, 20, 30, 40, 60 min, 2 h, 3 h, 6 h, 12 h, 24 h, after administration. In the third group, rats were administered intragastric gavage of *t*POA with 75 mg/kg body weight. Blood samples were collected at the time points of 0, 10, 20, 30, 40, 60 min, and 2 h, 3 h, 6 h, 12 h, 24 h. Serum was separated and stored frozen at − 80 °C until analysis. The following main pharmacokinetic parameters were analyzed using the non-compartmental pharmacokinetics data analysis soft-ware of PK solution 2 TM (Summit Research Service, Montrose, CO, USA): area under curve from zero to the last measurable serum concentration point (AUC_0–t_, t = 24 h), maximum concentration (Cmax), time-to-maximum concentration (Tmax).

## Results

### Determination of *c*POA and *t*POA by UPLC–MS/MS

**Figure 2:**
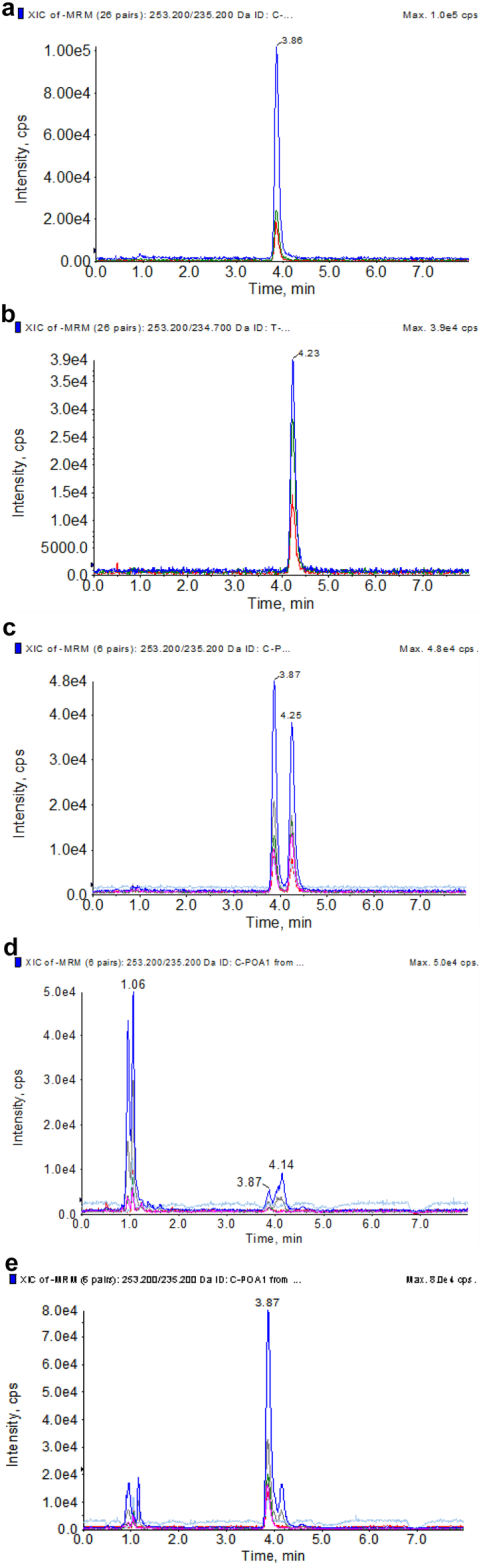
MRM chromatograms of *c*POA and *t*POA. (**a**) total ion chromatogram spiked with *c*POA standard sample, (**b**) total ion chromatogram spiked with *t*POA standard sample, (**c**) total ion chromatogram spiked with *c*POA and *t*POA standard sample, (**d**) blank rat serum sample, (**e**) rat serum sample after *c*POA intragastric gavage.

*c*POA and *t*POA were determined by ultra performance liquid chromatography ESI tandem mass spectrometry (UPLC–MS/MS). The total chromatography time was 8.0 min and retention time *c*POA and *t*POA were 3.86 min and 4.23 min, respectively (Fig. 2a–c). *c*POA and *t*POA were detected simultaneously in serum samples by intragastric administration after pretreatment. The results showed that the blank serum might contain trace amounts of *c*POA and *t*POA (Fig. 2d,e).

### Method validation

#### Selectivity and specificity

Selectivity and specificity were investigated by comparing chromatograms of 6 different blank rat serum samples to those of corresponding spiked serum samples. No other endogenous substances were observed to interfere with *c*POA and *t*POA in any samples, but trace amounts of *c*POA and *t*POA were observed in blanks. Specificity was verified by comparing retention times of *c*POA and *t*POA (3.86 and 4.23 min, respectively) in quality control (QC) samples (n = 6). The relative standard deviations of selectivity and specificity were less than 5%.

#### Calibration curve linearity, limit of detection (LOD) , and Lower limit of quantification (LLOQ)

Standard curves of *c*POA or *t*POA were obtained by plotting the ratios of peak areas of *associated Standard Solutions*. The curves were observed with correlation coefficients greater than 0.999 exhibiting good linearity over concentration ranges of 0.1–12 μg/mL for *c*POA and *t*POA. Typical calibration equations were y = 2.39e^+005x^ (R = 0.9991) for *c*POA, and y = 9.06e^+004x^ (R = 0.9999) for *t*POA, respectively, where y represented the peak area ratio of an analyte, and x represented an analyte concentration. The limit of detection (LOD) was estimated to be 30 ng/mL. The lower limits of quantification (LLOQ) for both *c*POA and *t*POA were defined as 0.1 μg/mL.

#### Accuracy and precision

Accuracy and precision of the present method were summarized in Table 2. Accuracy was required to be within ± 15% (20% for LLOQ) and precision not to exceed ± 15% (20% for LLOQ), according to the criteria for biological sample analysis suggested by U.S. Food and Drug Administration (FDA) guidelines^20^. Our results showed that the method was accurate and precise for simultaneous analysis of *c*POA and *t*POA in rat serum samples.

**Table 2:** Accuracy and precision for determination of *c*POA and *t*POA in serum samples (n = 6).

Analyte	Concentration (μg/mL)	Mean ± SD (μg/mL)	Accuracy (%)	Precision (%)
*c*POA	0.50	0.56 ± 0.15	111.0	0.98
	2.50	2.76 ± 0.86	110.4	1.45
	10.0	8.54 ± 1.79	85.40	2.01
*t*POA	0.50	0.54 ± 0.16	109.0	1.48
	2.50	2.64 ± 0.65	105.6	2.13
	10.0	8.15 ± 0.98	81.50	4.07

#### Recovery and matrix effects

The recoveries of *c*POA and *t*POA spiked into rat serum were determined at three QC levels. The recoveries of *c*POA were 101.43 ± 1.37, 102.11 ± 1.25, and 101.92 ± 1.84 (n = 6) at concentrations of 0.5, 2.5 and 10 μg/mL, and those of *t*POA were 98.28 ± 1.23, 100.66 ± 1.82, and 99.75 ± 3.01 (n = 6), respectively (Table 3). Matrix effects in the present study were investigated by a post-extraction spike method. The peak area of the standard analyte spiked into blank serum was compared with the corresponding peak area obtained were compared with that of injecting the standard analyte in the mobile phase at concentrations of 0.5, 2.5 and 10 μg/mL, all performed in triplicate. All the ratios of matrix effects defined above were within acceptable limits (89.51–94.72%) (Table 3). No significant matrix effect for *c*POA and *t*POA were observed, showing that ion suppression or enhancement from serum components was negligible for this method.

**Table 3:** Recovery and matrix effects of *c*POA and *t*POA in serum samples (n = 6).

Analyte concentration (μg/mL)	Matrix effects		Recovery	
	Mean ± SD (μg/mL)	RSD (%)	Mean ± SD (μg/mL)	RSD (%)
***c*POA**				
0.50	94.12 ± 1.91	2.96	101.43 ± 1.37	1.39
2.50	92.64 ± 1.67	1.98	102.11 ± 1.25	1.31
10.0	91.45 ± 2.43	2.56	101.92 ± 1.84	2.09
***t*POA**				
0.50	94.72 ± 1.05	1.27	98.28 ± 1.23	2.11
2.50	93.93 ± 2.47	2.69	100.66 ± 1.82	1.99
10.0	89.51 ± 3.05	3.55	99.75 ± 3.01	3.58

*RSD* relative standard deviation.

#### Stability

Results of stability tests indicated that the analytes were stable in laboratory conditions (Table 4). The stability of target compounds was evaluated as described in the experimental section.

**Table 4:** Stability of *c*POA and *t*POA (n = 6).

Storage condition (− 80 °C)	*c*POA			*t*POA		
	Concentration (μg/mL)	Accuracy (%)	RSD (%)	Concentration (μg/mL)	Accuracy (%)	RSD (%)
1 Freeze–thaw cycle	0.50	111.0	1.27	0.50	109.0	1.27
	2.50	110.4	1.53	2.50	105.6	1.53
	10.0	85.4	2.42	10.0	81.50	2.42
2 Freeze–thaw cycle	0.50	105.0	3.02	0.50	99.20	3.02
	2.50	107.6	2.98	2.50	101.4	2.98
	10.0	86.6	2.54	10.0	83.70	2.54
3 Freeze–thaw cycle	0.50	96.4	2.84	0.50	103.2	2.12
	2.50	99.2	3.07	2.50	91.70	2.97
	10.0	82.7	3.87	10.0	85.90	3.26

### Pharmacokinetic analyses of *c*POA and *t*POA

**Figure 3:**
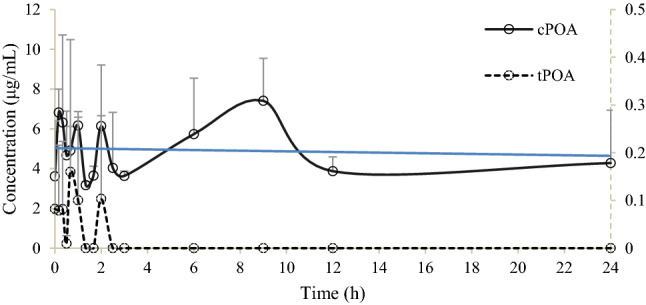
Mean serum concentration–time profiles of *c*POA and *t*POA in blank rat serum in 24 h (n = 6, mean ± SD). — shows mean serum concentration–time profiles of *c*POA, ---- shows mean serum concentration–time profiles of *t*POA.

The mean concentrations of *c*POA in blank group fluctuated around 5 μg/mL (4–8 μg/mL) in 24 h, while, those of *t*POA fluctuated around 2 μg/mL (0–4 μg/mL) in the beginning 2 h, and remained low after 2 h (Fig. 3), suggesting original *c*POA may exist in organism. The above observations also showed that the concentrations of *c*POA and *t*POA fluctuated in a similar trend in certain time.

**Figure 4:**
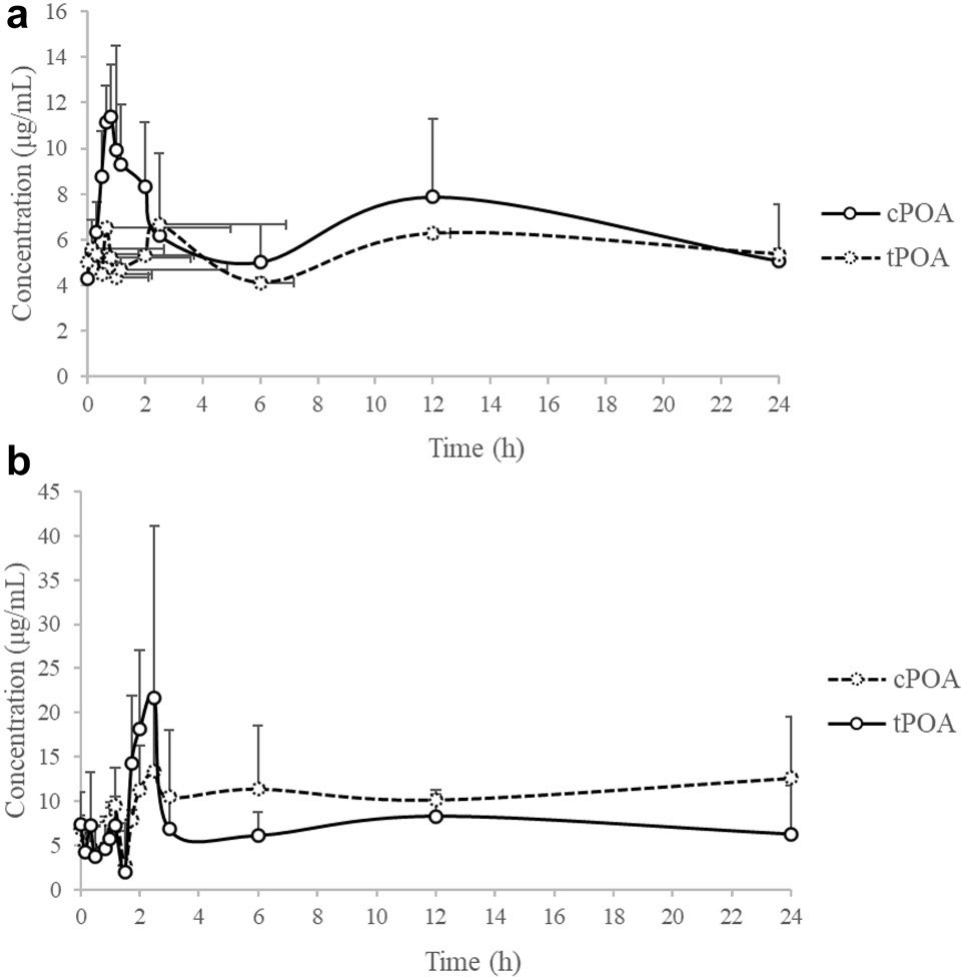
Mean serum concentration–time profiles of *c*POA and *t*POA (n = 6). (**a**) Showed *c*POA and *t*POA concentrations after i.g. administration of *c*POA in rats at 75 mg/kg body weight, (**b**) showed *c*POA and *t*POA concentrations after i.g. administration of *t*POA in rats at 75 mg/kg body weight.

The pharmacokinetic profiles of *c*POA and *t*POA were investigated by the treatment at 75 mg/kg i.g. dose of *c*POA to rats (Fig. 4a). The mean concentration–time profiles of *c*POA and *t*POA in serum showed that *c*POA was absorbed rapidly by rats and increased in serum in 0.3 h after administration and decreased slowly thereafter (Fig. 4a). The Tmax of *c*POA was 0.94 ± 0.44 h and the Cmax 8.17 ± 1.97 μg/L (Table 5). The concentration of *c*POA decreased to the blank level rapidly. Meanwhile, *t*POA was detected simultaneously after *c*POA dosing whose concentration–time profile fluctuated (4–7 μg/L) showed the same trend with *c*POA. And, the concentration of *t*POA in serum was retain 4–6 μg/L.

**Table 5:** Pharmacokinetic parameters of baseline-corrected *c*POA and *t*POA in rats after i.g. administration of *c*POA and *t*POA at 75 mg/kg body weight.

Parameters	Unit	Mean ± SD	
		*c*POA	*t*POA
C_max_	mg/L	8.17 ± 1.97	14.77 ± 11.91
T_max_	h	0.94 ± 0.44	1.50 ± 0.98
AUC (0–24 h)	mg/L*h	59.45 ± 29.83	113.88 ± 72.25

The pharmacokinetic profiles of *c*POA and *t*POA were investigated following a single i.g. dose at 75 mg/kg body weight of *t*POA to rats (Fig. 4b). The mean serum concentration–time profiles of *c*POA and *t*POA showed that *t*POA was absorbed slowly by rats such that it increased in serum in 2 h after administration and decreased rapidly thereafter (Fig. 4b). The Tmax of *t*POA was 1.50 ± 0.98 h and the Cmax 14.77 ± 11.91 μg/L. *t*POA was decreased to the concentration of original level rapidly. Meanwhile, *c*POA was detected after *t*POA dosing simultaneously, mean serum concentration–time profiles of *c*POA fluctuated (5–15 μg/L) in the same trend with *c*POA. And, the concentration of *c*POA in serum was maintain 10 μg/L, which was more than original concentration of *c*POA.

**Figure 5:**
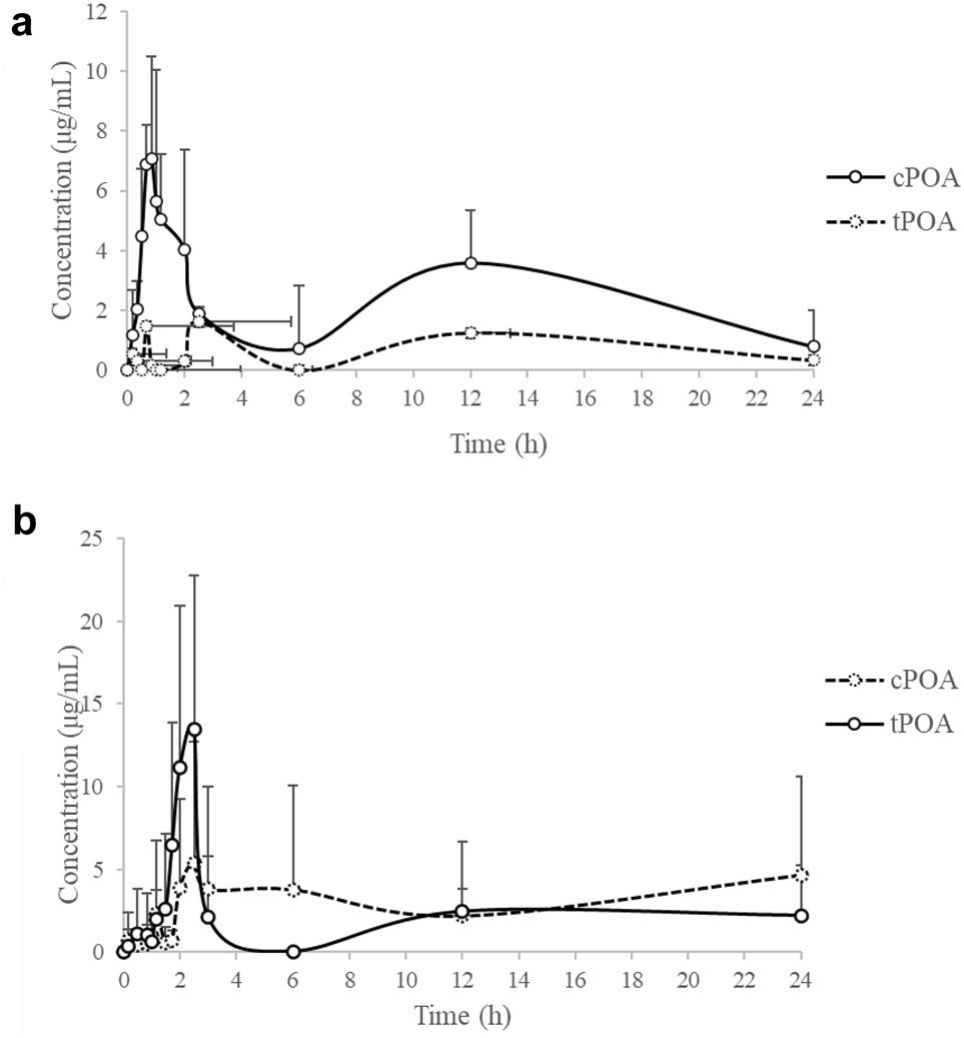
Baseline-corrected serum concentration–time profiles of *c*POA and *t*POA (n = 6). (**a**) Showed *c*POA and *t*POA concentrations after i.g. administration of *c*POA in rats at 75 mg/kg body weight, (**b**) showed *c*POA and *t*POA concentrations after i.g. administration of *t*POA in rats at 75 mg/kg body weight.

As mentioned above, pharmacokinetic analysis was made on *c*POA and *t*POA, both of which were with pre-existing endogenous levels (Fig. 3). Therefore, a correction based on the pre-existing baseline were necessary. Since the endogenous concentrations of *c*POA and *t*POA were variable, when discussing the total concentration of the two isomers, the contents of the inter- and intra-individual compounds were added some variabilities. So, a pre-dose adjustment of measured levels was conducted. For each rat at any test period, the pre-dose concentrations were subtracted from the measured serum concentrations, and the pharmacokinetic parameters was calculated on the baseline-adjusted concentrations according to regulatory guidelines for bioequivalence testing of endogenous substances^21,22^. If the measured value was lower than the pre-dose concentration, the adjusted one was set to zero (Fig. 5a,b).

Pharmacokinetic parameters including C_max_, T_max_ and AUC_0–24 h_ were determined using non-compartmental analysis according to the concentrations of *c*POA and *t*POA in the serum (Table 5). The area under the serum concentration–time curve (AUC) was calculated by applying the log-linear trapezoidal model to the measured concentrations of *c*POA, *t*POA and the combined sum of them at the actual sampling time points. Because of the fluctuated pre-existing endogenous levels, it was not possible to define a terminal elimination phase. The elimination rate constant was therefore not calculated, and exposure was described as the AUC from time 0 to 24 h post-dosing (AUC_0–24 h_). AUC_0–24 h_ of *c*POA and *t*POA were 59.45 ± 29.83 and 113.88 ± 72.25 mg/L·h.

## Discussion

*c*POA in organisms, especially in adipose tissue, may be related to the development of cardiovascular disease and *t*POA has been used as a biomarker for reducing the risks of type 2 diabetes and coronary heart disease^10–12^. The correlation between *c*POA to *t*POA may play a decisive role in related biological activities in the body.

We established a UPLC–MS/MS method with highly precision to detect trace amounts of *c*POA and *t*POA in animal serum, which did not require sample esterification pretreatment. Meanwhile, it was successfully used in the pharmacokinetic research, finding that the change of exogenous *c*POA concentration in serum accompanied with the fluctuation of *t*POA (endogenous) concentration with a consistent trend. Similarly, the change of *c*POA (endogenous) concentration in serum was also correspondent with the fluctuation of exogenous *t*POA concentration.

Huang et al. studied the effect of oral seabuckthorn fruit oil (main ingredient *c*POA, without *t*POA) on the content changes of *t*POA and *t*POA phosphatide fatty acids in fasting serum of healthy adults, and found that the content of *t*POA increased with high oral dose of *c*POA (1520 mg/day) for 3 consecutive weeks^23^. By simulating the differentiation of adipocytes in vitro, it was found that the increasing content of *t*POA would increase the contents of VA (18:1, *t*11) and conjugated linoleic acid (CLA, 18:2, *c*9, *t*11) in adipocytes^24^. *t*POA in the body was mainly from food intake and endogenous oxidation reaction. Isooleic acid (VA, 18:1, *t*11) in food could be converted into *t*POA (16:1, *t*9) by shortening the chain length, with a conversion rate of 17%^9^. As *t*POA in daily diet was with relatively low amount and easy to be oxidized, that in serum was mainly derived from endogenous transformation of VA from dietary^25^. Therefore, we speculated that *c*POA and *t*POA in organism may interconvert each other. Of course, this inference based on a comparative observation needed to be confirmed by further studies.

The pretreatment process of GC method was cumbersome and had a long total chromatography time and retention time. Establishing rapid, accurate and simultaneous detection of *c*POA and *t*POA in biological samples would benefit laboratorial and clinical research and real-time detecting *c*POA and *t*POA in organisms. Our method could be applied to clinical investigations to elucidate the biological activity and structure–activity relationship (SAP) of *c*POA and *t*POA in humans and favor the development of biological activity, drug delivery systems or regenerative medicine to define the metabolism and transformation between fatty acids. Besides, previous studies have shown that *t*POA was better than *c*POA in regulating lipid metabolism in hyperlipidemia mice^26^. Therefore, whether *c*POA played a role in regulating lipid metabolism by transforming into *t*POA, and the bioconversion relationship between *c*POA and *t*POA and the conversion rate could be further studied by isotope labeling or fluorescence labeling method.

## Conclusions

A sensitive and rapid UPLC–MS/MS method for determination of *c*POA and *t*POA simultaneously was established and used to identify them in rat serum after i.g. administration. Trace of isomers *c*POA and *t*POA could be quickly isolated and quantified in 5 min using this method. And it was further used in a pharmacokinetic study of *c*POA and *t*POA in rats. The results suggested that the *c*POA concentration in serum was correspondent with the fluctuation of *t*POA concentration indicating *c*POA and *t*POA in organism may interconvert each other.

## Data Availability

The data sets used and/or analyzed during the current study are available from the corresponding author on reasonable request.

## Abbreviations

POA: Palmitoleic acid
*c*POA: *Cis*-Palmitoleic acid
*t*POA: *Trans*-Palmitoleic acid
UPLC–MS/MS: Ultra performance liquid chromatography–tandem mass spectrometry

## References

[1] Morgan NG, Dhaya S (2010). Unsaturated fatty acids as cytoprotective agents in the pancreatic beta-cell. Prostag. Leukotr. Essent..

[2] Matthan NR, Dillard A, Lecker JL (2009). Effects of dietary palmitoleic acid on plasma lipoprotein profile and aortic cholesterol accumulation are similar to those of other unsaturated fatty acids in the F1B golden Syrian hamster. J. Nutr..

[3] Anoop M, Neha S, Lokesh K (2010). Obesity, the metabolic syndrome, and type 2 diabetes in developing countries: Role of dietary fats and oils. J. Am. Coll. Nutr..

[4] Souza CO, Teixeira AAS, Lima EA (2014). Palmitoleic acid (n-7) attenuates the immunometabolic disturbances caused by a high-fat diet independently of PPARα. Mediat. Inflamm..

[5] Bergman BC, Howard D, Schauer IE (2013). The importance of palmitoleic acid to adipocyte insulin resistance and whole-body insulin sensitivity in type1 diabetes. J. Clin. Endocrinol. Metab..

[6] Yang ZH, Takeo J, Katayama M (2013). Oral administration of omega-7 palmitoleic acid induces satiety and the release of appetite-related hormones in male rats. Appetite.

[7] Yang ZH, Miyahara H, Hatanaka A (2011). Chronic administration of palmitoleic acid reduces insulin resistance and hepatic lipid accumulation in KK-Ay Mice with genetic type 2 diabetes. Lipids Health Dis..

[8] Brouwer IA, Wanders AJ, Katan MB (2013). Trans fatty acids and cardiovascular health: Research completed. Eur. J. Clin. Nutr..

[9] Jaudszus A, Kramer R, Pfeuffer M (2014). Trans palmitoleic acid arises endogenously from dietary vaccenic acid. Am. J. Clin. Nutr..

[10] Mozaffarian D, de Oliveira Otto MC, Lemaitre RN (2013). Trans-palmitoleic acid, other dairy fat biomarkers, and incident diabetes: The multi-ethnic study of atherosclerosis (MESA). Am. J. Clin. Nutr..

[11] Mozaffarian D, Cao H, King IB (2010). Trans-palmitoleic acid, metabolic risk factors, and new-onset diabetes in US adults. Ann. Intern. Med..

[12] Nunes EA, Rafacho A (2017). Implications of palmitoleic acid (palmitoleate) on glucose homeostasis, insulin resistance and diabetes. Curr. Drug Targets.

[13] Brands M, Gutbrod P, Dörmann P (2021). Lipid analysis by gas chromatography and gas chromatography–mass spectrometry. Methods Mol. Biol..

[14] Fan YL, Arbab AAI, Zhang H (2021). MicroRNA-193a-5p regulates the synthesis of polyunsaturated fatty acids by targeting fatty acid desaturase 1 (FADS1) in bovine mammary epithelial cells. Biomolecules.

[15] Guillocheau E, Penhoat C, Gaëtan D (2020). Current intakes of trans-palmitoleic (trans-C16:1 n-7) and trans-vaccenic (trans-C18:1 n-7) acids in France are exclusively ensured by ruminant milk and ruminant meat: A market basket investigation—ScienceDirect. Food Chem. X.

[16] Zong G, Ye XW, Sun L (2012). Associations of erythrocyte palmitoleic acid with adipokines, inflammatory markers, and the metabolic syndrome in middle-aged and older Chinese. Am. J. Clin. Nutr..

[17] de Oliveira Otto MC, Lemaitre RN, Song XL (2018). Serial measures of circulating biomarkers of dairy fat and total and cause-specific mortality in older adults: The Cardiovascular Health Study. Am. J. Clin. Nutr..

[18] Pranger IG, Muskiet FAJ, Kema IP (2019). Potential biomarkers for fat from dairy and fish and their association with cardiovascular risk factors: Cross-sectional data from the LifeLines Biobank and cohort study. Nutrients.

[19] U.S. Food and Drug Administration, Guidance for Industry: Bioanalytical Method Validation. http://www.unodc.org/documents/scientific/validationE.pdf (2013).

[20] Bioanalytical method validation guidance for industry bioanalytical method validation. Center for Drug Evaluation and Research. FDA Guid. Ind., 1–22 (2013).

[21] FDA. Draft guidance on omega-3-acid ethyl esters (2017). https://www.fda.gov/downloads/drugs/guidancecomplianceregulatoryinformation/guidances/ucm320011.pdf. Accessed 1 July 2017.

[22] European Medicines Agency. Guideline on the investigation of bioequivalence (2010). http://www.ema.europa.eu/docs/en_GB/document_library/Scientific_guideline/2010/01/WC500070039.pdf. Accessed 1 July 2017. 10.1111/j.1742-7843.2009.00518.x 20070293

[23] Huang NK, Matthan NR, Galluccio JM (2020). Supplementation with seabuckthorn oil augmented in 16:1n–7t increases serum trans-palmitoleic acid in metabolically healthy adults: A randomized crossover dose-escalation study. J. Nutr..

[24] Kadegowda AKG, Burns TA, Miller MC (2013). Cis-9, trans-11 conjugated linoleic acid is endogenously synthesized from palmitelaidic (C16:1 trans-9) acid in bovine adipocytes. J. Anim. Sci..

[25] Guillocheau E, Garcia C, Gaëtan D (2019). Retroconversion of dietary trans-vaccenic (trans-C18:1 n-7) acid to trans-palmitoleic acid (trans-C16:1 n-7): Proof of concept and quantification in both cultured rat hepatocytes and pregnant rats. J. Nutr. Biochem..

[26] Huang WW, Hong BH, Bai KK (2020). Cis- and trans-palmitoleic acid isomers regulate cholesterol metabolism in different ways. Front. Pharmacol..

